# Choosing a Minister

**Published:** 1861-09

**Authors:** 


### CHOOSING A MINISTER.

When a congregation or parish becomes vacant, it is the dictate of the highest wisdom to select an incumbent at the earliest day practicable. In all vacancies, time is the fruitful fomenter of difference, discord, dissension, anarchy. A people without a stated preacher will wander away to hear various speakers, which is a fruitful source of differences of opinion; and different members of the same congregation, hearing the same preacher at different times, will disagree in their estimates of the same man, as no one is equally able on all occasions.

In making a choice, each one should cultivate a determination to abide by the will of the majority; and when a man is once fairly elected, stand by and sustain him, as if such choice were his own.

A compromising spirit should be sedulously cherished by every lover of "peace and concord." There should be an humble mistrust of one's own wisdom, and a becoming deference to that of others. Each one should tremble in view of taking the responsibility of a choice on himself, and say, "not as I will, but as ye;" thus generously sacrificing his own preferences to those of others. A minister selected with such feelings and sentiments, could scarcely fail to be a blessing to any community, the end being that all will come home as the day of life wanes, "bringing their sheaves with them."

